# Case report: Rare observation of thyroid-like cholangiocarcinoma

**DOI:** 10.3389/fmed.2024.1458586

**Published:** 2025-01-23

**Authors:** Ekaterina Bondarenko, Dmitriy Kalinin, Liliya Urusova, Dariya Pastukhova, Rustam Salimkhanov, Natalia Mokrysheva

**Affiliations:** ^1^Laboratory of Pathomorphology, Endocrinology Research Centre, Moscow, Russia; ^2^Department of Pathological Anatomy, National Medical Research Centre for Surgery Named after. A.V. Vishnevsky, Moscow, Russia; ^3^Department of Parathyroid Pathology and Mineral Disorders, Endocrinology Research Centre, Moscow, Russia; ^4^Endocrinology Research Centre, Moscow, Russia

**Keywords:** cholangiocarcinoma, liver, thyroid-like structure, immunohistochemistry, case report

## Abstract

Intrahepatic cholangiocarcinoma is a highly malignant tumor with a poor prognosis. Radical surgical resection remains the “gold standard” for improving patient outcomes; however, only a minority of patients qualify for this approach. Intrahepatic cholangiocarcinoma is primarily classified into two major histologic types: small and large ductal cholangiocarcinomas. Nevertheless, rare subtypes with unique diagnostic and prognostic characteristics are increasingly reported. These subtypes often exhibit features such as slow growth, a histologic architecture resembling thyroid tissue, or ductal ectasia, and are associated with a more favorable prognosis. We present the case of a 61-year-old patient with a solitary liver mass initially identified as a hemangioma through imaging studies. Histopathologic examination of the postoperative specimen revealed a thyroid-like structural pattern. Immunohistochemical analysis showed positive staining for CK7 and CK19, confirming the diagnosis of intrahepatic cholangiocarcinoma with a thyroid-like structure. The tumor was completely resected with clear margins, and no evidence of metastasis was found. Consequently, the patient was managed without adjuvant chemotherapy. At 14 months of follow-up, there were no signs of recurrence or metastasis. This clinical case underscores the importance of recognizing novel subtypes of cholangiocarcinoma and exercising vigilance in the management of patients with presumed benign hepatic lesions.

## Introduction

Cholangiocarcinoma is the second most common primary liver malignancy after hepatocellular carcinoma, accounting for approximately 15% of all primary liver tumors and 3% of all gastrointestinal cancers (1, 2). Despite its high morbidity and mortality rates, the clinical course of cholangiocarcinoma is highly variable (3). The tumor is classified into two main histologic types based on duct size: small-duct and large-duct cholangiocarcinomas (2, 4). Differentiating these types requires histopathologic, clinicopathologic, and molecular analyses. The most common subtypes include sclerosing, nodular, and papillary (polypoid) forms (5).

A particularly rare variant of intrahepatic cholangiocarcinoma exhibits histologic features resembling thyroid carcinoma. Fewer than 10 cases of this subtype have been reported globally. This rarity, coupled with its slow growth and histologic resemblance to thyroid tumors or ectopia, poses significant diagnostic challenges. Accurate diagnosis requires a thorough immunohistochemical (IHC) study and the exclusion of thyroid pathology.

## Clinical case description

A 61-year-old male patient presented with a 3-year history of right subcostal heaviness and belching. Based on clinical manifestations, a diagnosis of gastroesophageal reflux disease (GERD) with reflux esophagitis was established. Conservative therapy following standard protocols was initially effective; however, symptoms recurred after discontinuing the treatment.

**Figure 1:**
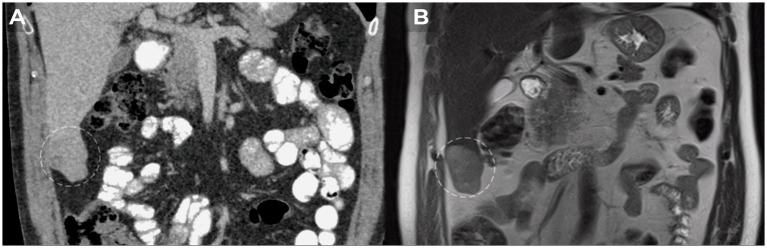
MRI dynamics of liver tumor growth. **(A)** Heterogeneous mass with clear irregular contours of 5×3 cm in the lower part of the 6th liver segment detected in 2021. **(B)** Growth dynamics of the liver tumor up to 7×5 cm in 2022.

In September 2021, hepatobiliary ultrasound (US) revealed a 54 × 30 mm mass in the 5th segment of the liver’s right lobe. Continued monitoring of the mass was recommended. By September 2022, contrast-enhanced magnetic resonance imaging (MRI) of the abdomen showed tumor growth to 70 × 42 × 50 mm, now occupying the 5th and 6th liver segments. The imaging characteristics suggested a vascular origin, such as hemangioma (Figure 1).

Due to progressive growth, surgical resection of the 5th and 6th liver segments was performed in January 2023. The postoperative period was uneventful.

Macroscopic examination of the resected specimen revealed a well-circumscribed subcapsular nodule measuring 7.5 × 3.0 × 4.0 cm, composed of dense, gray cystic tissue filled with transparent yellow colloid.

Histologic evaluation confirmed that the tumor consisted of well-differentiated tubular and glandular structures with focal cystic expansion and an abundance of eosinophilic, amorphous material within the lumen. Tumor cells appeared relatively uniform, cubic or cylindrical, with narrow cytoplasm and rounded nuclei. The tumor structures were set within a weakly hyalinized, edematous, desmoplastic stroma in certain areas. There was no evidence of invasive growth into the serosa or vascular invasion.

Given the unique architectural features, a secondary malignant neoplasm of the liver was initially suspected, raising the possibility of metastasis from thyroid carcinoma or renal carcinoma. However, thyroid ultrasonography revealed no abnormalities.

**Figure 2:**
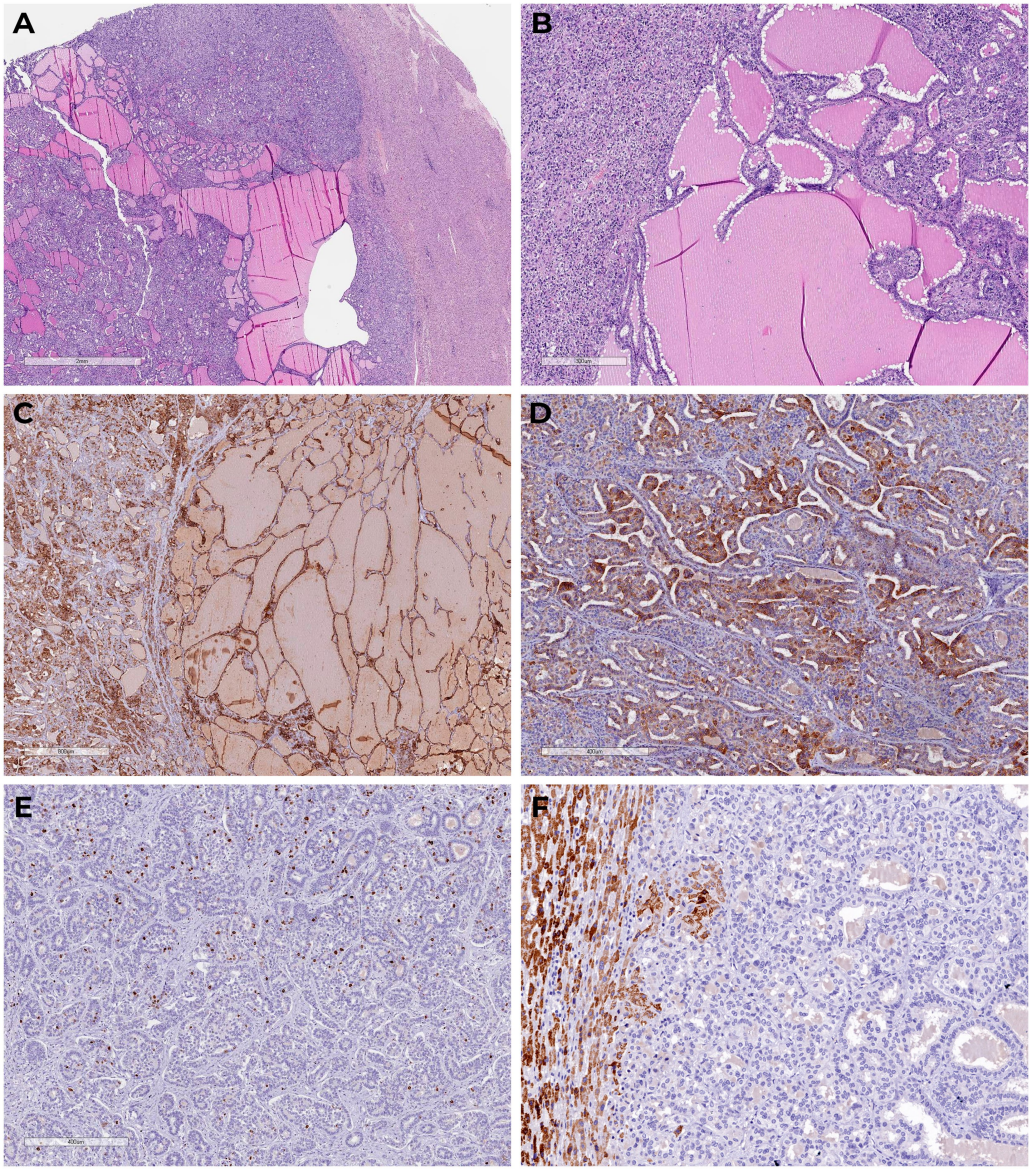
Tumor sections with glandular structures, cystic expansion, abundance of eosinophilic amorphous material in the lumen (H&E). **(A,B)** There is diffuse marked membranous cytoplasmic expression of cytokeratin 7 **(C)** and moderate focal membranous cytoplasmic expression of cytokeratin 19 **(D)** in the tumor cells; proliferative expression index by Ki-67 **(E)**; we detected granular cytoplasmic expression of TTF1 in the surrounding normal liver tissue, in the absence of primary thyroid cancer-specific nuclear expression in the tumor **(F)**.

Multistep IHC demonstrated moderate focal membrane-cytoplasmic expression of cytokeratin 19 (CK19, clone A53-B1/A2.26, Cell Marque) and diffuse, prominent membrane-cytoplasmic expression of cytokeratin 7 (CK7, clone OV-TL 12/30, Cell Marque). The proliferative index (Ki-67, clone MIB-1, DAKO) was 13.2% (75 of 569 cells). No expression was detected for thyroid transcription factor-1 (TTF1, clone 8G7G3/1, Cell Marque), inhibin alpha (clone R1, Cell Marque), CDX2 (clone DAK-CDX2, DAKO), CD56 (clone 123C3.D5, Cell Marque), chromogranin A (clone DAK-A3, DAKO), synaptophysin (clone MRQ-40, Cell Marque), or PAX-8 (polyclonal, Cell Marque) (Figure 2).

Based on histologic and IHC findings, a diagnosis of intrahepatic cholangiocarcinoma with a thyroid-like variant (pT1b, N0, cM0, Pn0, L0, V0, R0) was established.

### Follow-up

Dynamic surveillance without chemotherapy was recommended due to the absence of tumor foci in the resection margins, vascular invasion, and evidence of metastasis. At 14 months after the initial diagnosis, there were no signs of tumor recurrence. The patient reported satisfactory subjective quality of life.

## Discussion

The incidence of malignant neoplasms of the liver and intrahepatic bile ducts has risen significantly over the past decade. Despite advancements in diagnostic techniques and therapeutic approaches, the prognosis remains poor, with rising mortality rates, particularly among men (1, 6, 7). Surgical resection with histologic confirmation of negative margins remains the gold standard for treatment (2, 6). However, surgery often has a palliative intent and is frequently followed by chemotherapy, which is challenging due to the severity of the disease (8). According to the World Health Organization, nearly all intrahepatic bile duct neoplasms are adenocarcinomas, varying in their degree of differentiation (2, 9). Recent years have seen an increasing number of reports on rare variants of cholangiocarcinoma. These include spindle cell carcinomas, Epstein–Barr virus-positive lymphoepithelioma-like carcinomas resembling nasopharyngeal carcinoma (10), and cholangioblastic variants of cholangiocarcinoma (11, 12).

Intrahepatic cholangiocarcinoma resembling thyroid cancer, also known as thyroid-like cholangiocarcinoma, typically presents as a solitary, well-circumscribed nodule without evidence of serosal involvement. This variant is characterized by a relatively low Ki-67 proliferative index, absence of pathologic mitoses, and a desmoplastic stromal reaction, often complicating diagnosis. In some cases, these lesions are initially deemed benign. A detailed clinical history and thorough patient evaluation are crucial for establishing an accurate diagnosis. Most cases of thyroid-like cholangiocarcinoma described in the literature have demonstrated a favorable prognosis with long-term follow-up (Table 1). Tumor sizes reported in the literature range from 3 to 19 cm (Table 1).

**Table 1:** Clinical, morphologic and outcome reports of thyroid-like cholangiocarcinoma cases.

Authors	Sex	Age, years	Thyroid pathology	Tumor size, cm	Treatment	Immunohistochemistry		Follow-up period
						+	-	
(10)	Male	52	No	18	Surgical	СК7, СК19, CAM5.2, CK AE1	TTF1, thyroglobulin, СЕА, СК20, CD56, synaptophysin, chromogranin А, liver specific antigen	13 months without the disease recurring or metastasizing.
(13)	Female	26	No	19	Surgical & chemotherapy	CK7, CK19, CD138	TTF1, thyroglobulin, HepPar1, glypican-3, AFP, CD56, synaptophysin, chromogranin A	The patient died 28 months after diagnosis from recurrence and metastasis.
(14)	Male	59	No	3	Surgical	CK7, CK18, CK19, EMA, MUC1, CD10, glypican-3, p53, Ki-67, S100	TTF1, thyroglobulin, CD56, synaptophysin, chromogranin A, PAX8, CK20, CDX2, AFP, HepPar 1, CD34	16 months without the disease recurring or metastasizing.
(15)	Female	42	No	9	Surgical & chemotherapy	CK7, CK19	TTF1, HepPar1, AFP, CEA, CK20, CDX2, PAX8, synaptophysin	20 months without the disease recurring or metastasizing.
(3)	Male	60	Hashimoto’s thyroiditis	11	Surgical	CK7, synaptophysin (focal)	TTF1, arginase, chromogranin A	9 months without the disease recurring or metastasizing.
(16)	Female	23	Follicular variant of papillary microcarcinoma	12	Surgical	CK7, CK19	TTF1, thyroglobulin, PAX-8	18 months without the disease recurring or metastasizing.
(8)	Female	60	No	11	Surgical	CK7	TTF1 (8G7G3/1, Thermo Scientific), arginase (polyclonal, Sigma), hepatocyte-specific antigen (OCH1E5, Leica), glypican 3 (1G12, Leica), chromogranin A (LK2H10, Biogenex), synaptophysin	-
Our patient	Male	61	No	7,5	Surgical	CK19, CK7	TTF1, inhibin alpha, CDX2, CD56, сhromogranin A, synaptophysin, PAX-8	14 months without the disease recurring or metastasizing.

Morphologically, thyroid-like cholangiocarcinoma is composed of glandular structures with focal cystic expansion and abundant eosinophilic, amorphous material within the lumen. These features closely resemble metastases from thyroid cancer or ectopic thyroid tissue. While metastatic liver involvement in thyroid cancer is rare, occurring in less than 20% of cases, it is generally associated with widespread systemic disease (13, 14). Similarly, ectopic thyroid tissue in the liver is rare but can mimic a tumor or a primary multifocal neoplasm (15, 16).

## Conclusion

Our case represents the first reported instance of intrahepatic cholangiocarcinoma with thyroid-like morphology confirmed through IHC in a 61-year-old male. Over a 14-month follow-up period, there was no evidence of recurrence or metastasis. This case underscores the importance of expanding the classification of cholangiocarcinoma to include novel subtypes with unique morphologic criteria and clinical behavior. Recognizing such subtypes will enable more accurate diagnoses and closer surveillance of patients presenting with benign-appearing hepatic masses identified through imaging studies.

## Data Availability

The original contributions presented in the study are included in the article/Supplementary material, further inquiries can be directed to the corresponding author.
