# Supplementary figures and images for: Case report: Rare observation of thyroid-like cholangiocarcinoma
Source: Front Med (Lausanne). 2025 Jan 23;11:1458586. doi: 10.3389/fmed.2024.1458586 (PMC11799895; doi:10.3389/fmed.2024.1458586)

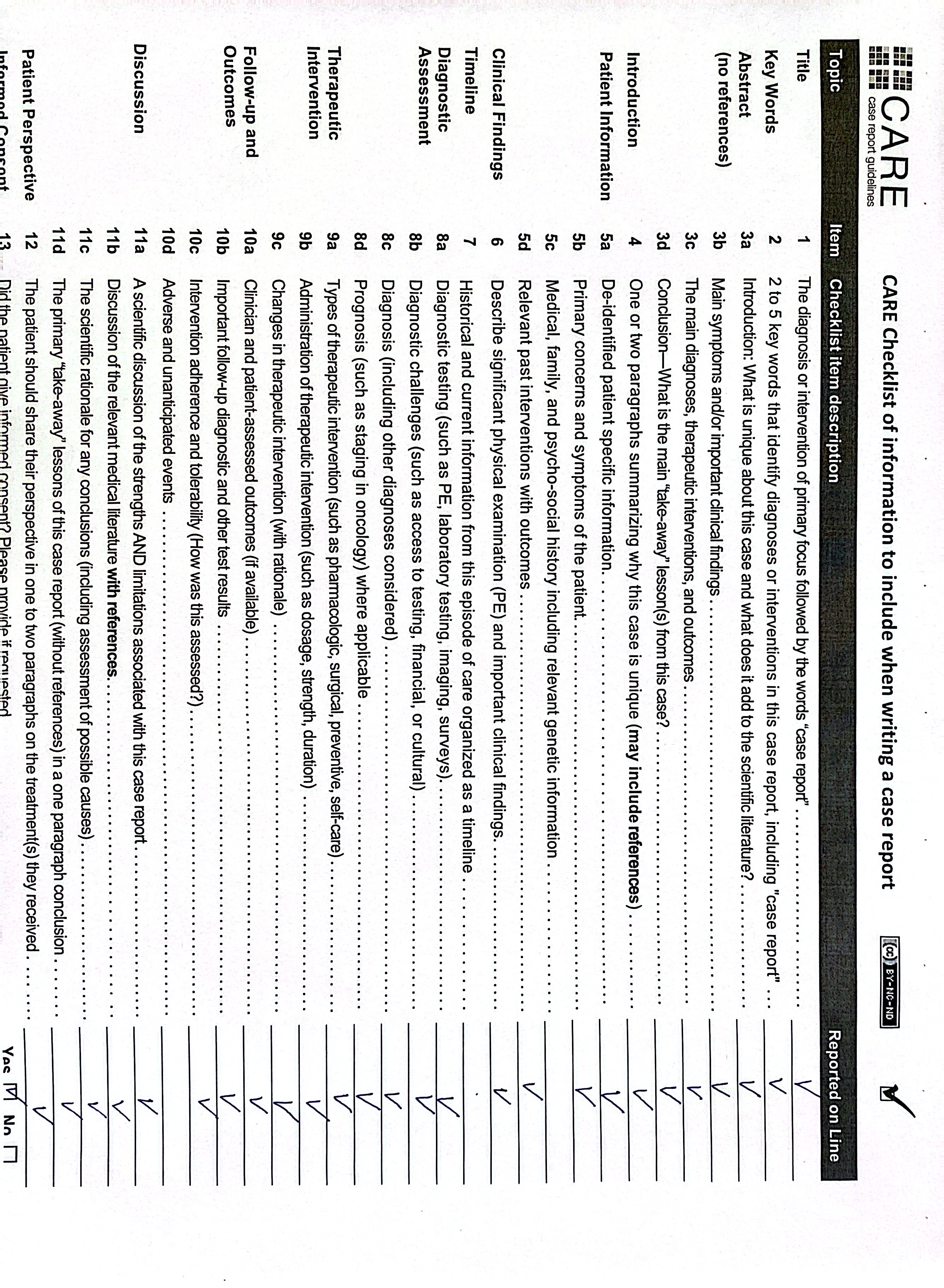

Supplement: Supplementary file 1 [file Image_1.JPEG]
